# Quality Improvement to Eliminate Disparities in Developmental Screening for Patients Needing Interpreters

**DOI:** 10.1097/pq9.0000000000000679

**Published:** 2023-08-07

**Authors:** Courtney M. Brown, Beth Dillon, Christina Toth, Emily Decker, Robin N. Alexander, Aarti R. Chandawarkar, Stefanie Bester, Elizabeth Ricket, Dane A. Snyder

**Affiliations:** From the * Division of Primary Care Pediatrics, Nationwide Children’s Hospital, Columbus, Ohio.; † Department of Pediatrics, The Ohio State University, Columbus, Ohio; ‡ Center for Clinical Excellence, Nationwide Children’s Hospital, Columbus, Ohio; § Center for Biostatistics, The Ohio State University, Columbus, Ohio.

## Abstract

Children from households with a preferred language other than English are less likely to receive timely identification and treatment for developmental delay than children of native English speakers. In dismantling this inequity, the role of primary care pediatrics is to establish equitable systems for screening and referral. This project, conducted in a network of twelve pediatric primary care centers, focused on eliminating a small but systematic disparity in developmental screening rates between families who did and did not require interpreters (86% versus 92%). The specific aim was to increase developmental screen completion among patients needing interpreters from 86% to 92% of age-appropriate well-child visits.

**Methods:**

Data were extracted from the electronic health record (EHR) to measure the proportion of 9-, 18-, 24-, and 30-month well-child visits at which developmental screens were completed, stratified by interpreter need (n = 31,461 visits; 7500 needing interpreters). One primary care center tested small changes to standardize processes, eliminate workarounds, and leverage EHR features using the Institute for Healthcare Improvement’s Model for Improvement. The QI team plotted screen completion on control charts and spread successful changes to all 12 clinics. Statistical process control evaluated the significance of changes in screening rates.

**Results:**

For patients needing interpreters, screen completion rose across all clinics from 86% to 93% when the clinics implemented the new process. Screen completion for patients not needing interpreters remained at 92%.

**Conclusion:**

A standardized process supported by the EHR improved developmental screening among patients needing interpreters, eliminating disparities.

## INTRODUCTION

Early identification and intervention are critical to optimizing outcomes for children with developmental delays. However, in the United States, children from households with a preferred language other than English (PLOE) are less likely to receive a diagnosis of developmental delay and less likely to receive developmental services before kindergarten than native English speakers.^1^ Young children from households with a PLOE face systematic disadvantages at every step of the developmental evaluation process. Compared with children from English-speaking homes, they are less likely to have health insurance,^2^ have a medical home,^3,4^ complete developmental screening in primary care,^5,6^ report experiencing family-centered care (through which discussions about developmental concerns may occur),^7^ and enroll in center-based childcare where screening may occur.^8^ Furthermore, when the developmental delay is identified, families with PLOE have more difficulty navigating referral systems than families whose preferred language is English.^9^ This gap in identifying and intervening for developmental delay is one of many examples of healthcare inequities experienced by this population that needs eliminating to achieve equitable health outcomes.^10,11^

In primary care, addressing developmental delay begins with screening. The American Academy of Pediatrics recommends developmental screening with standardized instruments at the 9-, 18-, 24-, and 30-month well-child visits.^12^ Improved developmental outcomes then hinge on successful connections to early intervention services. In the primary care network that conducted this study, a portfolio of quality improvement (QI) projects has been launched to ensure that children with suspected developmental delay receive timely and equitable access to developmental evaluations and therapies. This program’s initial QI work used standardization and technology (strategies already shown in the literature to be effective)^13^ to achieve a 90% screening rate at age-appropriate well-child visits. However, 30% of families in the network require interpreters, and those families received a developmental screening at systematically lower rates than those who did not require interpreters. Although the absolute difference between the two groups seem small (86% compared to 92%), control charts showed more variability and a systematically lower developmental screening rate for children whose families required interpreters. This manuscript describes a quality improvement project to address that disparity.

The specific aim was to increase the developmental screening rate among patients requiring interpreters to at least 92% (equivalent to the screening rate among patients not requiring interpreters) by June 2021 and sustain the change for six months.

## METHODS

### Context

This study was conducted by a network of 12 pediatric primary care centers affiliated with a Midwestern academic children’s hospital. The network provides primary care for >130,000 children, 74% enrolled in Medicaid. Thirty percent require interpreters for their visits. The network uses the Epic electronic health record (EHR, Epic Systems, Verona, WI). Preferred language and the need for an interpreter are entered into the EHR by registration staff, who undergo training to ascertain this information. Interpreter services are available in person, via or via video conferencing. The most common preferred languages among the patient population are English (66%), Spanish (14%), Somali (8%), and Nepali (4%).

Since November 2019, the primary care network has administered the developmental milestones section of the Survey of Well-Being of Young Children (SWYC) at 9-, 18-, 24-, and 30-month well-child visits. The SWYC is given to families at registration to complete in the waiting room. Initially, families completed the screen on paper, and providers or staff manually entered the responses into the EHR. In January 2020, the network began administering the SWYC on electronic tablets, automatically importing responses into the EHR. However, the SWYC was only available in English on the tablets, and the screening process was not standardized for patients who could not complete the SWYC in English. Some sites distributed translated versions of the SWYC on paper when translations were available, and staff then entered the responses manually into the EHR. At times, intake staff asked the SWYC questions via an interpreter. Others deferred to providers to perform developmental surveillance.

### Intervention

**Fig. 1.**
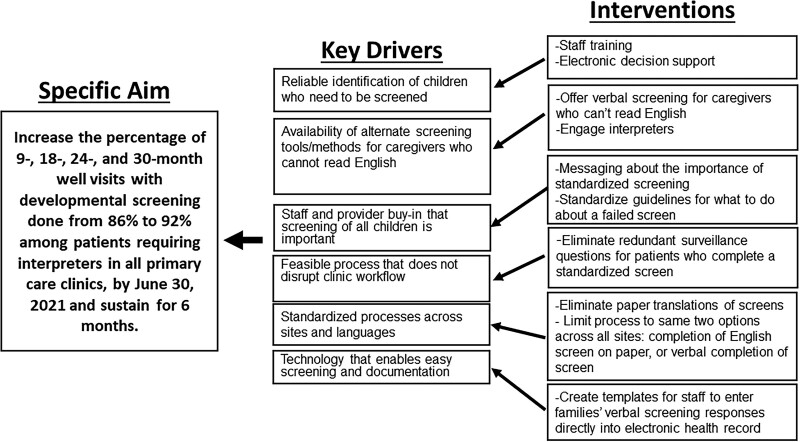
Key driver diagram.

The team leading the development and testing of the intervention included a primary care physician with advanced training in QI methods, the lead nurse at the primary care site with the largest proportion of patients needing interpreters, three physicians and one nurse with expertise in informatics, a quality improvement specialist, and the section chief for the primary care network. This team reviewed baseline data and developed a key driver diagram for improving developmental screening rates (Fig. 1).

The team then developed an intervention to standardize and streamline the developmental screening process for patients requiring interpreters. The new, standardized process eliminated paper translations of the screening forms. (Due to software and informatics workforce limitations, translated versions of the SWYC could not be added to the tablets.) Instead, intake staff asked the SWYC questions verbally with assistance from an interpreter. The intake staff entered the parents’ responses directly into a template in the EHR. To reduce the time spent on patient intake and increase the feasibility of performing the screen verbally, we eliminated redundant developmental surveillance questions from the intake script for young children’s well-child visits. The EHR system automatically imported SWYC responses into providers’ progress note templates, prompting providers to review the responses.

### Study of the Intervention

The QI team used the Institute for Healthcare Improvement’s Model for Improvement to test the intervention.^14^ The team conducted Plan-Do-Study-Act (PDSA cycles) to test small changes at one primary care clinic (the site with the largest percentage of patients requiring interpreters). At this test clinic, the QI team monitored the proportion of visits with SWYC screening completed each week among patients who required interpreters and those who did not. In addition, the QI team reviewed each test of change and asked clinic nurses for informal feedback on the benefits and drawbacks of each process change. Clinic staff also provided contextual information, such as whether one of the clinic’s regular nurses had been absent on a day the clinic tested a change. The QI team also examined SWYC completion among subgroups of visits, such as those occurring on evenings and weekends when regular staff were not in the clinic and adapted interventions for those scenarios. Once SWYC completion was equal between patients at the test clinic who did and did not require interpreters, preparations began to spread changes across the entire primary care network, with continued monitoring of weekly screening rates stratified by the need for an interpreter. Strategies to support spread to other clinics included discussing the new processes at staff meetings, EHR changes to guide staff through the new screening process, and training new staff in the process at orientation.

### Measures

The primary outcome measure was the proportion of 9-, 18-, 24-, and 30-month well-child visits with documentation of a completed SWYC in the EHR. The team defined SWYC completion as an answer entered into the EHR for all SWYC questions. Answers could be entered into EHR flowsheets by clinic staff or entered on tablets by parents and automatically imported into the EHR. The denominator for each month included all completed 9-, 18-, 24-, and 30-month well-child visits at all 12 primary care sites. Because the outcome was more likely to be influenced by office processes than by individual patient characteristics, we used the visit as the unit of analysis, not the patient. Neither the numerator nor the denominator included canceled or missed appointments. The analysis did not exclude the visit if the same patient had another visit earlier in the study. Unique patients may have had more than one well-child visit within the study period but not within a month. Study team members manually reviewed a sample of charts to check the validity of the SWYC completion data, ensuring that billing codes or free text notes did not contradict the report used for the analysis. The analyst separated the data into two groups: families’ who needed an interpreter and families who did not. This classification was based on the value entered by the registration staff in the EHR’s “need for interpreter” field. This field is distinct from a “Preferred Language” field.

A secondary measure was the percentage of completed SWYCs scoring in the “needs review” range. The team defined these ranges based on score cutoffs established by the SWYC developers.

### Analysis

The team’s analyst extracted outcome data from the EHR and plotted data monthly on control charts. Rational subgrouping was used to examine the pre-intervention difference between the two groups (families needing interpreters and those not). Data for each group were also plotted chronologically to examine the temporal relationships between shifts in the data and intervention implementation and/or other contextual events. The team set a priori rules based on statistical process control to identify significant changes in the process.^15^ Specifically, the team shifted the centerline if any of the following conditions was met: (1) eight or more consecutive points above or below the centerline; (2) six consecutive points increasing or decreasing; (3) two out of three consecutive points in the outer one-third of the chart close to the control limits; (4) fifteen consecutive points in the inner one-third of the chart close to the centerline; (5) fourteen points in a row alternating up and down; (6) special cause identified by the Aggregate Point Rule.^16^ This study met the institutional review board’s definition of “not human subjects research.”

## RESULTS

From March 2020 to November 2021, the primary care network conducted 31,461 total 9-, 18-, 24-, and 30-month well-child visits. Of these, 7500 required interpreters. During the pre-intervention period (March 2020 to March 2021), the overall proportion of visits requiring an interpreter with a completed SWYC documented was 84.5%. Conversely, the overall proportion of visits not requiring an interpreter with a completed SWYC documented was 90.2%.

**Fig. 2.**
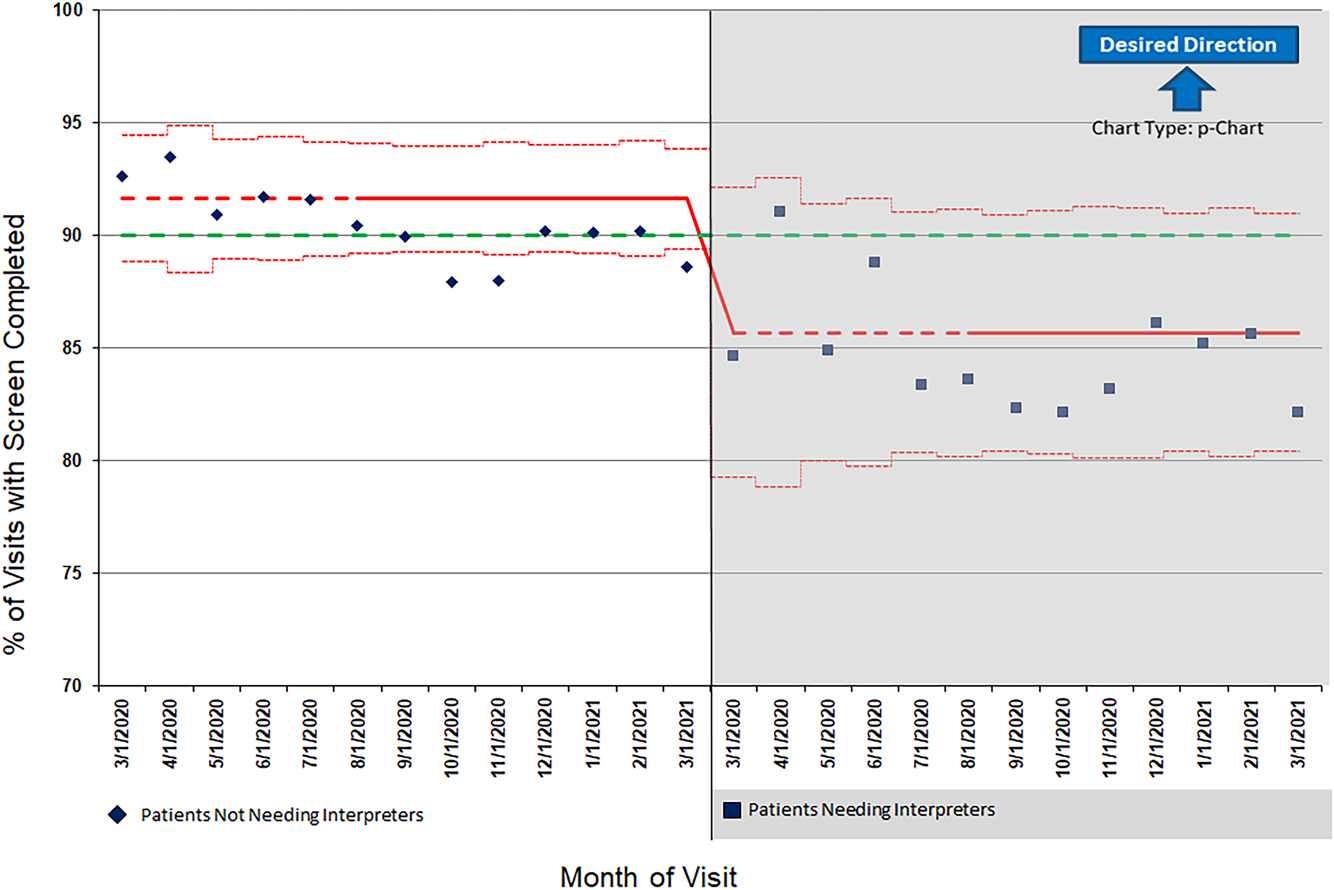
Baseline monthly percentage of 9-, 18-, 24-, and 30-month well-child visits with screen completion using statistical process control and rational subgrouping.

**Fig. 3.**
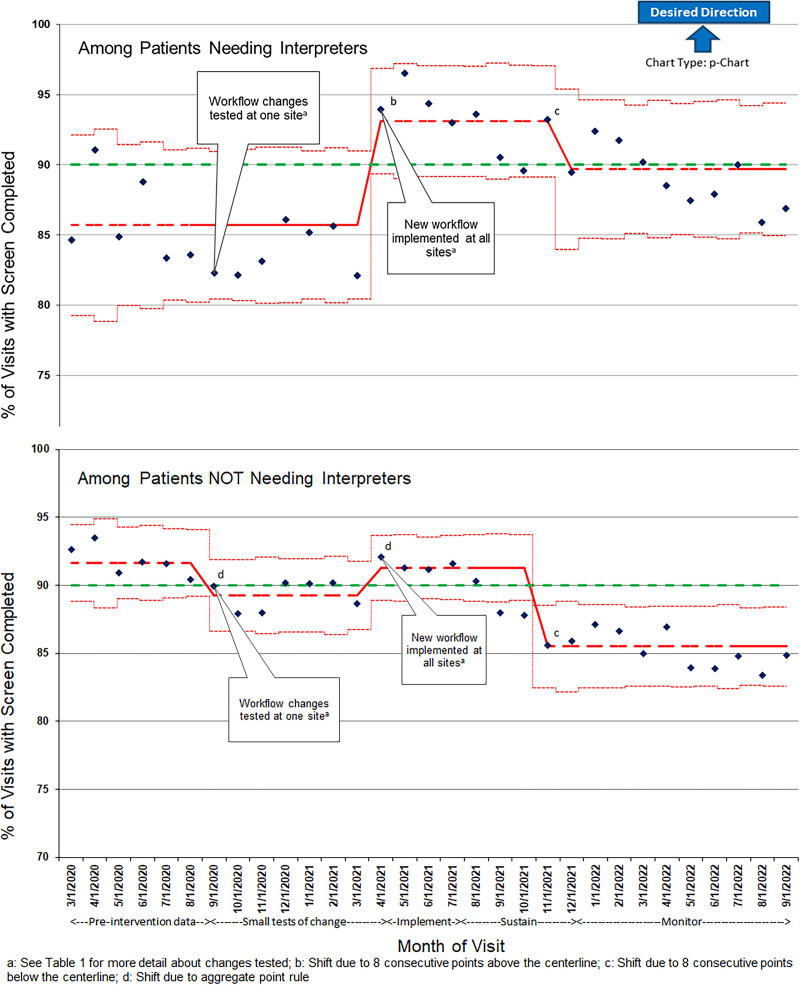
Monthly percentage of 9-, 18-, 24-, and 30-month well child visits with screen completion using statistical process control.

Figure 2 shows pre-intervention screening rates. The centerline is lower for visits requiring interpreters than those not requiring interpreters. The control limits are also wider for visits requiring interpreters, indicating a more variable process. The annotated control charts in Figure 3 show pre- and post-intervention screening rates, based on statistical process control. (Table 1 supplements the control chart annotations and describes the timeline of PDSA cycles in further detail.) For visits requiring interpreters, SWYC completion rose across all centers from 86% to 93% when the new process was implemented. The control limits also narrowed in this group, indicating less variability in screening rates with the new process. SWYC completion for visits not needing interpreters showed narrow control limits throughout the entire study period; the mean declined slightly from the pre-intervention to the testing period, then returned to 91% after the intervention was implemented at all sites. Based on statistical process control analysis, the mean percentage of SWYCs scoring as “needs review” among patients requiring interpreters decreased from 20% pre-intervention to 15% post-intervention.

**Table 1.** Process Changes Tested

Dates	Sites	PDSA Ramp	Driver(s)	What Was Learned
9/1/2020–10/1/2020	Test clinic	Pre-clinic huddles to identify patients with interpreters who need screening; nurses asked screening questions verbally using an interpreter.	Reliable identification of children who need to be screened; availability of alternate screening methods	Screens were completed more reliably with this method; confusion occurred when cross-covering registration or nursing staff present.
10/1/2020–11/15/2020	Test clinic	Disposed of paper translations of screening tools	Standardized processes	Improved screening rates on weekdays; variability on weekends (some still printed out translations or skipped screening)
11/15/2020–1/31/2021	Test clinic	Trained staff (including those who worked less frequently) to limit the process to two options—tablet in English or verbal with an interpreter	Standardized processes	This process resulted in less confusion and better screening rates but was time-consuming
1/31/2021–4/1/2021	All clinics	No active testing—prepared electronic health record; deleted redundant questions from intake process; trained staff	Staff and provider buy-in; feasible process; technology that enables easy screening and documentation	Staff and providers supportive of proposed changes
4/1/2021–11/30/2021	All clinics	Implemented new processes, monitored data, and re-trained staff as needed at lower-performing clinics	Standardized processes	Screening rates among patients requiring interpreters improved and were sustained

Informal feedback from staff indicated that the new standardized process had decreased uncertainty about handling developmental screening among patients who required interpreters and that the new EHR templates accommodated an all-digital process. However, staff indicated that the new screening process was more time-consuming for patients requiring interpreters.

Due to the COVID-19 pandemic, other contextual elements affected workflow during and after the study period. First, patient volumes and patient needs changed. The monthly number of well-child visits for children 9- to 30-months-old remained consistent throughout the study period, but the overall burden on the clinic from other patient needs fluctuated. From March 2020 to June 2021, the clinics saw fewer patients per day as the institution’s COVID-19 protocols limited the number of scheduled well-child visits for older children. Normal scheduling resumed in July 2021. From the fall of 2021 through all of 2022, the clinics faced new demands, including surges in ill visits from the delta and omicron waves of the COVID-19 pandemic,^17^ rising prevalence of mental health concerns,^18^ new COVID-19 vaccines to discuss and administer,^19^ and formula shortages to help families navigate.^20^ Following national trends,^21^ the primary care network also experienced fluctuations in staffing due to illness and resignations, requiring new hires across all roles. Because of this turbulence in the primary care setting, the study team continued to monitor the primary outcome measure for 6 months after the pre-defined study period to understand the sustainability of the interventions. SWYC completion decreased among both groups of patients (those requiring and not requiring interpreters), but completion rates among patients needing interpreters remained higher than those who did not (Fig. 3).

## DISCUSSION

This QI project eliminated a disparity in developmental screening rates for children whose families required interpreters. Although the pre-intervention disparity may seem small (86% versus 92%), screening rates were systematically lower for children whose families required interpreters: an example of the institutional practices that contribute to poorer health outcomes for children from groups that have been historically marginalized.^22^ When the primary care network confronted these differences in care and applied quality improvement methods to the problem, the network eliminated the disparity in screening rates. Successful strategies included standardization and an entirely EHR-based process rather than a mix of electronic and paper screening methods.

The study team had anticipated that the involvement of a live professional interpreter might affect how families answered the SWYC questions. After implementing the new process, the proportion of completed SWYCs scoring “needs review” decreased among patients requiring interpreters. It is unclear whether this change represents more false negatives (perhaps due to social desirability bias) or fewer false positives (due to the opportunity to ask a live interpreter to clarify the questions). The study team suggests two future intervention areas to ensure optimal identification of children at risk for developmental delay. First, it is important to make these translations available on tablets for languages in which validated translations of developmental screening tools exist. Second, empirical research is needed to validate parent-completed written screening tools in additional languages. Despite the practical advantages of written screening tools, many families likely struggle to complete them, even in their native languages. For families with low literacy or those who speak primarily oral languages (such as Somali), there is a need to study the validity of screening tools when verbally administered.

Surprisingly, after the intervention, rates of SWYC completion among patients requiring interpreters surpassed those of patients not requiring interpreters. Some families who did not require interpreters may have been unable to complete the screens on tablets due to literacy barriers or distractions.^23^ Most practices ask families to self-complete written screens for feasibility reasons.^24^ Still, our findings suggest written screening processes may disadvantage other patient groups beyond those with a PLOE, including those with low literacy or those who can speak but not read English. As suggested by health literacy experts, healthcare providers should suspect low literacy when patients fail to complete written forms and should offer help in a shame-free way.^25^ The primary care network intends to test strategies for more systematically offering such help to families regardless of their preferred language.

Of course, verbal screening introduces feasibility concerns. Unfortunately, this study could not quantify the intervention’s effects on visit duration or office workflow. The clinics could not feasibly collect data on how long it took to administer the screen verbally, and the study team could not use overall visit duration as a meaningful balancing measure because too many other factors influenced it. Staff reported that, subjectively, even with other modifications to the patient intake process, the new screening process took longer than if families completed translated developmental screening forms on paper. However, the Civil Rights Act mandates interpreter services, and using professional interpreters improves patient satisfaction and outcomes.^26^ Interestingly, after the study concluded and the clinics faced multiple pandemic-related challenges, screening rates declined equally in patients with and without interpreters. This fact suggests an enduring commitment to equitable care, even during time pressures. It also points to a broader concern that the pandemic has exacerbated primary care’s long-standing struggle to follow expansive preventive care recommendations.^27^ The necessary response is to bolster systemic supports that facilitate more reliable preventive care delivery for all patients in the post-pandemic environment. In the short term, this project team will continue to monitor developmental screening rates and explore additional strategies for efficient and effective communication with families to ensure that disparities do not re-emerge.

The study had additional limitations. Individuals may have been represented more than once in the data set. Although the data were collected over time, the data set comprises a distinct cross-sectional sample of individuals at each time point, and serial autocorrelation is not a concern. Also, other organizations may not be able to replicate these interventions. This study’s health system may have greater access to interpreter services and EHR customization than smaller practices. As a result, different settings may need different interventions.

Notably, screening is just the first step in addressing developmental delay. Providers must also refer children with delays for appropriate therapies, and families must access those therapies. Beyond the actions of the health care system, social factors influence access to therapy and families’ abilities to practice developmental skills at home. Like many other health outcomes,^28^ societal changes are needed to eliminate disparities in developmental delay.

Furthermore, this project focused on reducing disparities in care at the visit level. The project did not measure or address screening rates at the patient level or population level. Therefore, disparities in longitudinal measures (such as the percentage of patients who have completed four SWYCs by three years of age) might persist due to barriers to well-child visit attendance. Our institution works toward more equitable well-child visit attendance through separate family outreach, care coordination, and provider continuity initiatives.

A society committed to achieving equitable healthcare cannot accept systemic differences in care. By identifying a disparity and using QI methods to address it, this team improved developmental screening rates among patients requiring interpreters, exceeding that of patients who did not require interpreters. The next steps include (1) ensuring appropriate and equitable referrals for children identified with developmental concerns, (2) identifying patients who do not require interpreters but who may need or prefer a verbal screening method, (3) adding validated translations of screens to tablets, and (4) identifying and addressing any language-based disparities in other types of screens we perform on tablets (eg, maternal depression screening).

## ACKNOWLEDGMENT

We thank Sara Conroy and Benjamin R. Baer for their helpful guidance and thoughtful discussions.

## DISCLOSURE

The authors have no financial interest to declare in relation to the content of this article.
